# Risk of high blood pressure in salt workers working near salt milling plants: A cross-sectional and interventional study

**DOI:** 10.1186/1476-069X-4-13

**Published:** 2005-07-25

**Authors:** Kripa Ram Haldiya, Murli Lal Mathur, Raman Sachdev, Habibulla N Saiyed

**Affiliations:** 1 Deputy Director Senior Grade, Desert Medicine Research Centre (ICMR), Jodhpur, 342005, India; 2 Deputy Director, Desert Medicine Research Centre (ICMR), Jodhpur, 342005, India; 3 Deputy Director, Desert Medicine Research Centre (ICMR), Jodhpur, 342005, India; 4 Director, National Institute of Occupational Health (ICMR), Meghani Nagar, Ahmedabad, 380816, India

## Abstract

**Background:**

Workers working close to salt milling plants may inhale salt particles floating in the air, leading to a rise in plasma sodium, which, in turn, may increase the blood pressure and the risk of hypertension.

**Methods:**

To test the above hypothesis, occupational health check-up camps were organized near salt manufacturing units and all workers were invited for a free health examination. The workers who worked with dry salt in the vicinity of salt milling plants were defined as "non-brine workers," while those working in brine pans located far away from milling plants were defined as "brine workers." Blood pressure (BP) was measured during each clinical examination. In all, 474 non-brine workers and 284 brine workers were studied.

**Results:**

Mean systolic blood pressure of non-brine workers (122.1 ± 13.3 mm Hg) was significantly higher than that of brine workers (118.8 ± 12.8 mm Hg, p < 0.01). Mean diastolic blood pressure of non-brine workers (71.5 ± 10.4 mm Hg) was significantly higher than that of brine workers (69.7 ± 9.4 mm Hg, p = 0.02). The prevalence of hypertension was significantly higher in non-brine workers (12.2%) than in brine workers (7.0%, p = 0.02). Nineteen salt workers were monitored while they used face masks and spectacles, for six days. Systolic, as well as diastolic, blood pressure of these workers began declining on the third day and continued to decline on the fourth day, but remained stationary up to the sixth day. The concentration of salt particles in the breathing zone of these workers was 376 mg/m^3 ^air.

**Conclusion:**

Inhalation of salt particles in non-brine workers may be an occupational cause of increased blood pressure.

## Introduction

There is an abundance of scientific evidence demonstrating a direct relation between salt intake and blood pressure (BP) [1]. Many animal studies [2], large population-based studies [1,3-6], epidemiological studies [7-9], meta-analyses of clinical trials [10-12], and randomized controlled trials [13,14] have shown that BP is directly related to salt intake. People's occupations also have varying impact on their BP [15-19]. Salt workers involved in the process of manufacturing, milling, and packing of salt are exposed to salt via their environment. Since most salt milling plants in India are not fully enclosed, salt particles float in the air in the vicinity of the workers. These workers may therefore inhale considerable amounts of salt during working hours. These salt particles may be inhaled and therefore absorbed in the airway surface epithelium [20-24] or the lungs [25]. These same fine particles are also able to translocate from the lungs into the circulatory system [26]. Inhaled salt particles may be carried via a continuous upward mucocilliary current on the airway surface to throat, where they can be swallowed. This is likely to increase the plasma sodium level, which in turn may increase the BP [27] and the risk of hypertension in the exposed workers. However, this problem may be completely preventable. This hypothesis was tested through a cross-sectional and experimental study involving salt workers; the results are presented and discussed below.

## Methods

A cross-sectional study was conducted among salt workers of the Sambhar, Nawa, and Rajas salt-manufacturing sites of Rajasthan, which are at about 150 km. from Jaipur, the capital of Rajasthan. Occupational health check-up camps were held at these three sites, under the Project on Prevention and Control of Occupational Health Hazards Among the Salt Workers, sponsored by the Ministry of Health, Government of India. This project was approved by the Scientific Advisory Committee of the National Institute of Occupational Health, Ahmedabad, India. The procedures followed were in accordance with the Helsinki Declaration. The camps were organized at Sambhar, Nawa, and Phalodi, in collaboration with owners of salt manufacturing units and the Department of Salt, Government of India. Each camp lasted 5 days. All the workers from nearby salt manufacturing units were invited for a free health examination. Workers who were absent on the dates of the health camp were not included in the study.

The aim of the study was explained to the subjects. Their age (in years), sex, detailed occupational history (including exact nature of job and duration of working in salt industry) were recorded on schedules especially designed for occupational health examinations.

After obtaining the informed consent, the clinical examination was carried out by one of the authors, who did not measure the blood pressure. After the subject had rested for five minutes in a supine position, the blood pressure was measured in the right arm using digital blood pressure equipment (Omron T-4). The cuff size was 25 cm × 13 cm. Three readings were taken by the trained field investigators, under supervision of another author. The first two readings were to familiarize the subjects with the process and the third reading was recorded for analysis. Prior to the camps, the field investigators were trained by the authors for fifteen days in measuring blood pressure. Body weight and height were measured by another trained field investigator. Height was measured in centimeters, using an anthropometric rod, while the subject stood erect on a flat platform.

Eight-hundred-and-ninety-one salt workers attended the camps, and three blood pressure measurements were taken in 875 workers. The workers who were involved in crushing, grinding, milling, packing, and loading salt, and who did not work with brine, were defined as non-brine workers. These workers worked in the vicinity of salt milling plants. Workers who worked with brine pans for the purpose of crystal reshuffling and raw salt heaping were defined as brine workers; their site of work was far away from the salt milling plants. The workers who worked as non-brine workers for some time and also worked as brine workers on some other days were excluded from analysis. Workers who were involved in only administrative and other related activities were also excluded from the analysis.

Hypertension was defined as systolic blood pressure more than 139 mmHg and/or diastolic blood pressure 90 mmHg or above. Body mass index was calculated as [Weight in Kg/(Height in meters)^2^]. Systolic and diastolic blood pressure was compared in the brine workers and non-brine workers. Student's t-test and Chi square test were used to determine the statistical significance of the differences.

Since mean systolic BP, mean diastolic BP, and prevalence of hypertension were found to be significantly higher in non-brine workers as compared to brine workers, an intervention study was carried out to test the hypothesis that exposure of non-brine workers to salt particles floating in environment may contribute to rise in their blood pressure. For this purpose, thirty-three non-brine workers, working at or close to salt milling plants, who volunteered to participate in the study, were registered. We explained the study hypothesis and provided them with face masks and spectacles with plain glasses. The masks were dust guards made of poly vinyl chloride, containing a disposable filter cartridge of nitrocellulose. In one of our earlier studies, we found that these masks could filter 82.8% dust particles of size 10 μm or less [28]. The workers were trained and motivated to use them properly while working, and were observed and followed for six consecutive days. During this period, their resting blood pressure was measured in the supine position, before starting work in the morning. Only nineteen of them regularly attended the worksite and used the face mask and eyeglasses for all six consecutive days, while others were present on some days, but absent on others. Workers were requested to provide urine samples before starting the intervention study and after completing the intervention for more than 3 days. These samples were collected twice a day – once in the morning before starting work and then in the evening, after completion of working hours. Only eight subjects out of 33 workers provided both (morning and evening) urine samples before intervention and only six workers (not the same) provided both urine samples after intervention. These samples were then analyzed for sodium and potassium levels using an AVL electrode electrolyte analyzer (AVL Medical Instruments, Schaffhausen, Switzerland). Additionally, the concentration of salt particles in the air in the environment of the work site was measured by using a respirable dust sampler (Environtech). The dust sampler was placed at two sites namely Sambhar Salts and at Nawa for six days. The particles of 10 μm or more were collected at the bottom of the cyclone of the sampler and those smaller than 10 μm were deposited on the filter paper of the sampler. Volume of total air entering the sampler and weight of particles collected were used to calculate the average concentration of both types of dust in the environment.

## Results

Out of 758 salt workers studied, 474 (62.5%) workers were non-brine workers, while 284 (37.5%) were brine workers. The characteristics of the study subjects are depicted in Table 1. These were comparable in brine and non-brine workers. Mean age of male brine workers was 31.8 ± 9.8 years, while male non-brine workers were comparatively a little younger (mean age 29.2 ± 10.0 years.). The mean age of female brine workers (35.1 ± 10.9 years) was not significantly different from that of female non-brine workers (36.5 ± 10.5 years). All workers were 15 years of age or older. The two groups did not show any significant difference in the prevalence of smoking, alcohol use, literacy, income, diet habits, and BMI. However, mean duration of working in the salt industry was lower in non-brine workers than brine workers.

**Table 1:** Characteristics of study subjects.

**Characteristics**	**Brine workers (n = 284)**	**Non-brine workers (n = 474)**	**p value**
**Age (Years)**			
Males	31.8 ± 9.8 (n = 238)	29.2 ± 10.0 (n = 398)	<0.01*
Females	35.1 ± 10.9 (N = 46)	36.5 ± 10.5 (N = 76)	0.49†
Both Sexes	32.3 ± 10.0	30.4 ± 10.4	0.01*
**Gender **M/F (%)	83.8/16.2	84.0/16.0	0.97 ‡
**Literacy (%)**	35.2	43.5	0.03 ‡
**Income (Rs. per anum)**	17760.9 ± 12858.7	19684.5 ± 13761.4	0.06†
**Smokers (%)**	33.8	35.9	0.25 ‡
**Alcohol users (%)**	10.6	11.8	0.17 ‡
**BMI Kg/m2**	18.9 ± 2.2	18.7 ± 2.5	0.28†
**Vegetarians (%)**	62.3	67.7	0.15 ‡
**Duration of working in salt industry (Years)**	11.4 ± 7.2	8.7 ± 6.9	<0.01*

*Difference significant; Student's t-test

† Difference not significant; Student's t-test

‡ Difference not significant; Chi Square test

Mean systolic blood pressure of non-brine workers (122.1 ± 13.3 mmHg) was significantly higher than that of brine workers (118.8 ± 12.8 mm Hg)(p < 0.01). Z-test, as well as the Student's t-test (two-tailed), showed a highly significant difference in both sexes, separately (Table 2). Mean diastolic blood pressure of non-brine workers (71.5 ± 10.4 mm Hg) was significantly higher than that of brine workers (69.7 ± 9.4 mm Hg) (p = 0.01). This was also consistently higher in both sexes.

**Table 2:** Mean systolic and diastolic blood pressure of brine workers and non-brine workers.

	**Brine workers**	**Non-brine workers**	**p value**
**Average systolic BP**			
Males	119.9 ± 11.7 (n = 238)	122.8 ± 12.4 (n = 398)	<0.01*
Females	113.2 ± 16.6 (n = 46)	118.3 ± 16.5 (n = 76)	0.01
Both sexes	18.8 ± 12.8 (n = 284)	122.1 ± 13.3 (n = 474)	<0.01*
**Average diastolic BP**			
Males	69.4 ± 9.6 (n = 238)	72.8 ± 10.2 (n = 398)	0.09
Females	71.1 ± 7.8 (n = 46)	75.2 ± 10.3 (n = 76)	0.02*
Both sexes	69.7 ± 9.4 (n = 284)	71.5 ± 10.4 (n = 474)	0.01*

*Difference significant; Z-test and Student's t-test (two-tailed)

Overall, the prevalence of hypertension in salt workers was 10.3%. It was significantly higher in non-brine workers (12.2%) than in brine workers (7.0%) (p = 0.02). The prevalence of hypertension was also consistently higher in non-brine workers than brine workers in different groups, according to age, sex, literacy, income, and body-mass index, duration of working in salt industry, smoking, alcohol use, tobacco chewing, and diet (Table 3).

**Table 3:** Prevalence of hypertension in brine workers and non-brine workers according to various characteristics.

**Characteristics**	**Brine Workers**			**Non-brine Workers**		
	
	**No.**	**Hypertensive cases**		**No.**	**Hypertensive cases**	
		**No.**	**%**		**No.**	**%**
**Age **<40 years	208	11	5.3	368	33	9.0
40+ years	76	9	11.8	106	25	23.6
Males	238	18	7.6	398	46	11.6
Females	46	2	4.3	76	12	15.8
**Illiterate**	184	16	8.7	268	38	14.2
**Literate**	100	4	4.0	206	20	9.7
**Annual income **Rs.<18000	179	10	5.6	256	33	12.9
>18000	105	10	9.5	218	25	11.5
**BMI **<18 Kg/m^2^	109	3	2.8	185	18	9.7
18+ Kg/m^2^	175	17	9.7	289	40	13.8
**Duration of Work **<10 Years	118	5	4.2	305	31	10.2
10+ Years	166	15	9.0	169	27	16.0
**Smokers or ex-smokers**	190	15	7.9	316	43	13.6
**Non-smokers**	94	5	5.3	158	15	9.5
**Alcohol **Users or ex-users	259	17	6.6	430	55	12.8
Non-users	25	3	12.0	44	3	6.8
**Tobacco chewing **Yes	217	14	6.5	325	40	12.3
No	67	6	9.0	149	18	12.1
**Diet **Vegetarian	177	15	8.5	321	41	12.8
Mixed	107	5	4.7	153	17	11.1
**Total Prevalence**	**284**	**20**	**7.0**	**474**	**58**	**12.2**

### Results of experimental intervention

**Figure 1:**
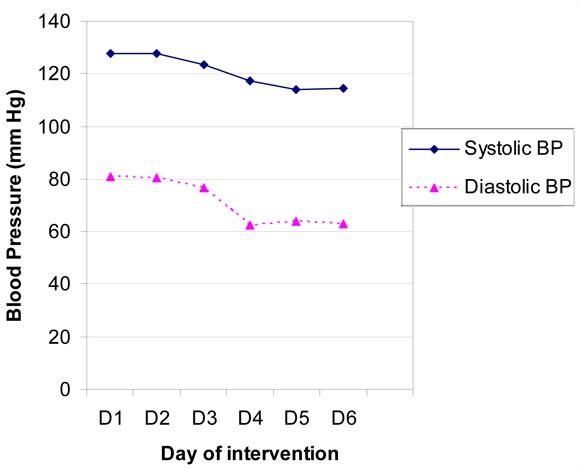
Blood Pressure of non-brine workers declined by use of masks and glasses.

Table 4 shows the mean number of working hours, mean number of hours for which masks and glasses were used, and the mean morning blood pressure of nineteen workers who attended the worksite and used face mask and eyeglasses for all six days of intervention. Morning blood pressure was taken before starting their shift. The systolic, as well as diastolic, blood pressure of these workers began declining on the third day and continued to decline on forth day, but remained stationary, each day thereafter (Figure 1). Table 5 shows that the difference in blood pressure between day 1 and day 2 was not significant (for systolic BP p = 0.98 and for diastolic BP p = 0.95), but that between day 2 and day 3 (for systolic BP p = 0.03 and for diastolic BP p = 0.16), as well as between day 3 and day 4, was significant (for systolic BP p = 0.03 and for diastolic BP p < 0.01); again, the decline thereafter was not significant (for systolic BP p = 0.08 & 0.68 and for diastolic BP p = 0.55 & 0.65). Mean urinary sodium in morning samples before the intervention was 265.7 ± 250.8 mmol/L and decreased to 184.6 ± 46.3 mmol/L three days after the intervention. This decline was not statistically significant (p = 0.27). Mean urinary sodium in evening samples before the intervention was 310.8 ± 304.2 mmol/L, as compared to 180.5 ± 41.2 mmol/L three days after the intervention. This decline was also not statistically significant (p = 0.31). Mean concentration of salt particles of a size less than 10 μm (PM 10) was 15 mg/m^3 ^and that of larger particles was 361 mg/m^3 ^air in the breathing zone of these workers, during these six days.

**Table 4:** Mean working hours, period of use of protective devices and morning blood pressure of workers on the days of intervention (n = 19).

Day of intervention	Mean no. of hours Worked	Mean no. of hours masks used	Mean no. of hours glasses used	Mean Systolic Blood Pressure (mm Hg)	Mean Diastolic Blood Pressure (mm Hg)
Day 1	6.2 ± 0.5	3.9 ± 1.0	4.8 ± 0.8	127.8 ± 11.1	80.7 ± 8.8
Day 2	10.0 ± 1.4	5.5 ± 1.5	6.5 ± 1.6	127.8 ± 11.8	80.6 ± 12.8
Day 3	9.7 ± 1.8	4.6 ± 1.3	5.2 ± 1.9	123.4 ± 10.3	76.4 ± 8.6
Day 4	7.9 ± 0.5	4.4 ± 1.9	4.8 ± 1.3	117.5 ± 9.9	62.6 ± 7.8
Day 5	9.3 ± 0.9	4.2 ± 1.1	4.5 ± 0.9	113.8 ± 7.0	63.8 ± 8.0
Day 6	9.1 ± 1.3	4.8 ± 1.4	5.2 ± 1.5	114.6 ± 6.5	63.0 ± 5.5
**Total**	**8.7 ± 1.7**	**4.6 ± 1.5**	**5.1 ± 1.5**		

**Table 5:** Comparison of mean systolic and diastolic blood pressures of the workers on consecutive days of intervention.

Day of intervention	Mean Systolic Blood Pressure (mm Hg)	p value	Mean Diastolic Blood Pressure (mm Hg)	p value
Day 1	127.8 ± 11.1	0.98	80.7 ± 8.8	0.95
Day 2	127.8 ± 11.8		80.6 ± 12.8	
				
Day 2	127.8 ± 11.8	0.04*	80.6 ± 12.8	0.16
Day 3	123.4 ± 10.3		76.4 ± 8.6	
				
Day 3	123.4 ± 10.3	0.03*	76.4 ± 8.6	0.001*
Day 4	117.5 ± 9.9		62.6 ± 7.8	
				
Day 4	117.5 ± 9.9	0.08	62.6 ± 7.8	0.55
Day 5	113.8 ± 7.0		63.8 ± 8.0	
				
Day 5	113.8 ± 7.0	0.68	63.8 ± 8.0	0.65
Day 6	114.6 ± 6.5		63.0 ± 5.5	

* Difference significant; Student's t-test (two-tailed)

## Discussion

In the present study, systolic, as well as diastolic, BP and prevalence of hypertension were found to be higher in non-brine salt workers, who were occupationally exposed to sodium chloride particles in the air of the breathing zone. This is a new observation, though it is in line with the hypothesis that, after being inhaled, salt may be absorbed from respiratory tract [20-24] or the mucocilliary current may transport it to pharynx, where it is swallowed and can then be absorbed from the gastrointestinal tract. Consequent increases in plasma sodium may be responsible for increases in the BP [27]. Differences in urinary sodium, an indicator of sodium intake, and plasma sodium are associated with BP differences of clinical and public health relevance [29]. The exact mechanisms whereby raised plasma sodium increases the BP are not clear. Existing concepts focus on the tendency for an increase in extracellular fluid volume (ECV), but raised plasma sodium increases a transfer of fluid from the intracellular to the extracellular space, and stimulates the thirst center. Accordingly, the rise in plasma sodium is responsible for the tendency for an increase in ECV. Although the change in ECV may have a pressure effect, the associated rise in plasma sodium itself may also cause the BP to rise [27]. Systolic and diastolic BP and the prevalence of hypertension of the non-brine (exposed) workers were compared with the brine salt workers, who were not exposed to salt particles in air. BP is affected by multiple factors, including age, nature of job, socioeconomic status, living standard, nutritional status, smoking habits, and alcohol consumption. Both groups of studied workers did not differ on these parameters (Table 1). However, mean age and mean duration of working in the salt industry (exposure) were lower in non-brine workers, compared to brine workers, but these can be causes of lower BP, rather than of higher BP. The prevalence of hypertension was consistently higher in different subgroups of non-brine workers (Table 3), and this consistency further strengthens the above observation. It can, therefore, safely be concluded that BP and prevalence of hypertension of non-brine workers were higher than brine workers.

To further confirm the hypothesis about probable mechanism involved, an experimental intervention was carried out. The decline in BP while using face masks and spectacles during work again strengthens this hypothesis. The urinary sodium levels also declined after use of masks and glasses for three days, though the decline was not statistically significant (probably because of smaller sample size).

A limitation of the study is that serum sodium levels of the workers involved in the intervention study could not be measured. The total concentration of salt particles in the air was 376 mg/m^3^. Considering the average tidal volume of 800–1000 ml/breath and respiratory rate of 18–25/min while working, the average worker could inhale 2.60 to 4.51 gm sodium chloride over the course of an eight hour shift. Average use of the mask was 52.9% of working hours, which could have prevented inhalation of about 1.37 to 2.39 gm of salt per day. The exact mechanism by which the decline of 1.37 to 2.39 g of salt intake per day could significantly reduce the BP is not clear. Thus, the results of this intervention study do not fully support the hypothesis that the cause of higher BP and higher prevalence of hypertension in non-brine workers is inhalation of salt particles from the environment. Eye glasses were provided to protect their eyes from salt particles and the study design did not allow us to find out whether these contributed to lowering of BP, though the salt particle sticking on to conjunctiva may also pass along with tears through the naso-lacrimal duct to respiratory tract and further on to the gastrointestinal tract. The psychological effect of using some of the intervention devices expected to reduce BP can also not be ruled out in this study. Further studies on salt workers are needed to elucidate our findings.

## List of Abbreviations

BP : Blood Pressure

ECV : Extra Cellular fluid Volume

## Competing interests

The author(s) declare that they have no competing interests.

## Authors' contributions

KRH contributed in conception and design, acquisition of data, analysis and interpretation of data and drafting the paper; MLM contributed in acquisition of data, statistical analysis and interpretation of data and drafting the paper; RS contributed in acquisition of data and drafting the paper; and HNS contributed in conception and design and critical evaluation of the data and drafting of the paper. All authors read and approved the final manuscript.
